# Proteasome and heat shock protein 70 (HSP70) inhibitors as therapeutic alternative in multiple myeloma

**DOI:** 10.18632/oncotarget.22815

**Published:** 2017-12-01

**Authors:** Angela Isabel Pereira Eugênio, Veruska Lia Fook-Alves, Mariana Bleker de Oliveira, Rodrigo Carlini Fernando, Daniela B. Zanatta, Bryan Eric Strauss, Maria Regina Regis Silva, Marimélia Aparecida Porcionatto, Gisele Wally Braga Colleoni

**Affiliations:** ^1^ Discipline of Hematology e Hemotherapy, Department of Clinical and Experimental Oncology, Universidade Federal de São Paulo, UNIFESP, São Paulo, SP, Brazil; ^2^ Center of Translational Investigation in Oncology, Cancer Institute of the State of São Paulo, Faculdade de Medicina, Universidade de São Paulo, São Paulo, SP, Brazil; ^3^ Department of Pathology, Universidade Federal de São Paulo, UNIFESP, São Paulo, SP, Brazil; ^4^ Department of Biochemistry, Universidade Federal de São Paulo, UNIFESP, São Paulo, SP, Brazil

**Keywords:** multiple myeloma, HSP70, ubiquitin-proteasome system, unfolded protein response, autophagy

## Abstract

HSP70 connects multiple signaling pathways that work synergistically to protect tumor cells from death by proteotoxic stress and represents a possible target to establish a new approach for multiple myeloma treatment. Therefore, bioluminescent cell lines RPMI8226-LUC-PURO and U266-LUC-PURO were treated with HSP70 (VER155008) and/or proteasome (bortezomib) inhibitors and immunodeficient mice were used for subcutaneous xenograft models to evaluate tumor growth reduction and tumor growth inhibition after treatment. Bioluminescence imaging was used to follow tumor response. Treatment with bortezomib showed ∼60% of late apoptosis in RPMI8226-LUC-PURO (without additional benefit of VER155008 in this cell line). However, U266-LUC-PURO showed ∼60% of cell death after treatment with VER155008 (alone or with bortezomib). RPMI8226-LUC-PURO xenograft presented tumor reduction by bioluminescence imaging after treatment with bortezomib, VER155008 or drug combination compared to controls. Treatment with bortezomib, alone or combined with VER155008, showed inhibition of tumor growth assessed by bioluminescence imaging after one week in both RPMI8226-LUC-PURO and U266-LUC-PURO cell lines when compared to controls. In conclusion, our study shows that the combination of proteasome and HSP70 inhibitors induced cell death in tumor cells *in vitro* (late apoptosis induction) and *in vivo* (inhibition of tumor growth) with special benefit in U266-LUC-PURO, bearing 17p deletion.

## INTRODUCTION

Multiple myeloma (MM) is a lymphoid malignancy that represents 1% of all cancer cases and 13% of hematological neoplasms and still remains an incurable disease [1]. Even with an aggressive therapy, including immunomodulatory drugs (thalidomide, lenalidomide, pomalidomide), proteasome inhibitors (bortezomib, carfilzomib) and autologous transplant, survival of high risk MM patients is still poor [2–5]. Cytogenetics is one of the most important prognostic factors in MM and, according to current guidelines, patients with at least one of the following features at diagnosis, such as deletion of 17p, t(14;16) or t(14;20) detected by FISH analysis, have high risk MM [6].

Felix *et al.* (2009) [7] identified differentially expressed genes in normal and MM patient’s plasma cells using Serial Analysis of Gene Expression (SAGE) and real time PCR (qPCR) validation. One of these genes, *P53CSV/TRIAP1 (TP53* regulated inhibitor of apoptosis 1), was overexpressed in more than 90% of MM cases. *TRIAP1* activates apoptotic pathways through interaction with HSP70, preventing APAF-1 (apoptosis protease activating factor 1), cytochrome *c*, and caspase-9 apoptosome complex formation [8, 9]. Overexpression of HSP70 may provide a selective advantage for tumor cell survival due in part to its ability to inhibit cell death through APAF-1 and caspase 9 [10].

HSP70 activates protein homeostasis, one of the essential mechanisms for myeloma cell survival, avoiding tumor death caused by intracellular accumulation of abnormal immunoglobulin, also known as monoclonal component [11]. Thus, some metabolic pathways, including HSP70 and its members, ubiquitin-proteasome pathway and cellular stress pathways, such as unfolded protein response (UPR) and autophagy, will contribute to the ability of neoplastic cells to adapt to the stress caused by immunoglobulin overload in the endoplasmic reticulum (ER). Since these pathways are used by tumor cells to ensure their survival, they can also be considered attractive therapeutic targets [12–14].

The HSP70 family is composed by 13 members of highly conserved proteins. The members better known for their cellular functions are: HSPA1A and HSPA1B (referred together as HSP70 or HSP72), HSPA5 (also known as BIP), HSPA8 (also known as HSC70), and HSPA9. They are involved in protein homeostasis and synthesis, folding of misfolded proteins (HSPA1A, HSPA1B an HSPA5), solubilization of protein aggregates, protein degradation through proteasome pathway and autophagy (HSPA1A, HSPA1B and HSPA8) [10, 15].

Evidences show that *HSP70* is overexpressed in many types of cancer and that high levels of this chaperone are linked with high tumor grade and/or poor prognosis. Inhibition of HSP72 and HCS70 simultaneously by shRNAs silencing resulted in retention of light chain immunoglobulins in myeloma cell lines (RPMI-8226 and KMS-11), causing proteotoxic stress and interfering with cell growth and survival [11, 16, 17, 18].

Since several studies have proved that tumor cells (and not normal cells) present high levels of HSP70, which is involved in several mechanisms of protein homeostasis, the use of a HSP70 inhibitor could make tumor cells more sensitive to proteasome inhibitors and prevent the functioning of the proteasome and related pathways [10, 19].

Clinical studies have tested HSP90 inhibitors combined with bortezomib in MM, but there is no evidence of clinical trials using HSP70 inhibitors in combination with bortezomib. [20] Therefore, the aim of this study is to explore the potential of HSP70 as a target in MM, through *in vitro* and *in vivo* analyses using proteasome and HSP70 inhibitors.

## RESULTS

**Figure 1:**
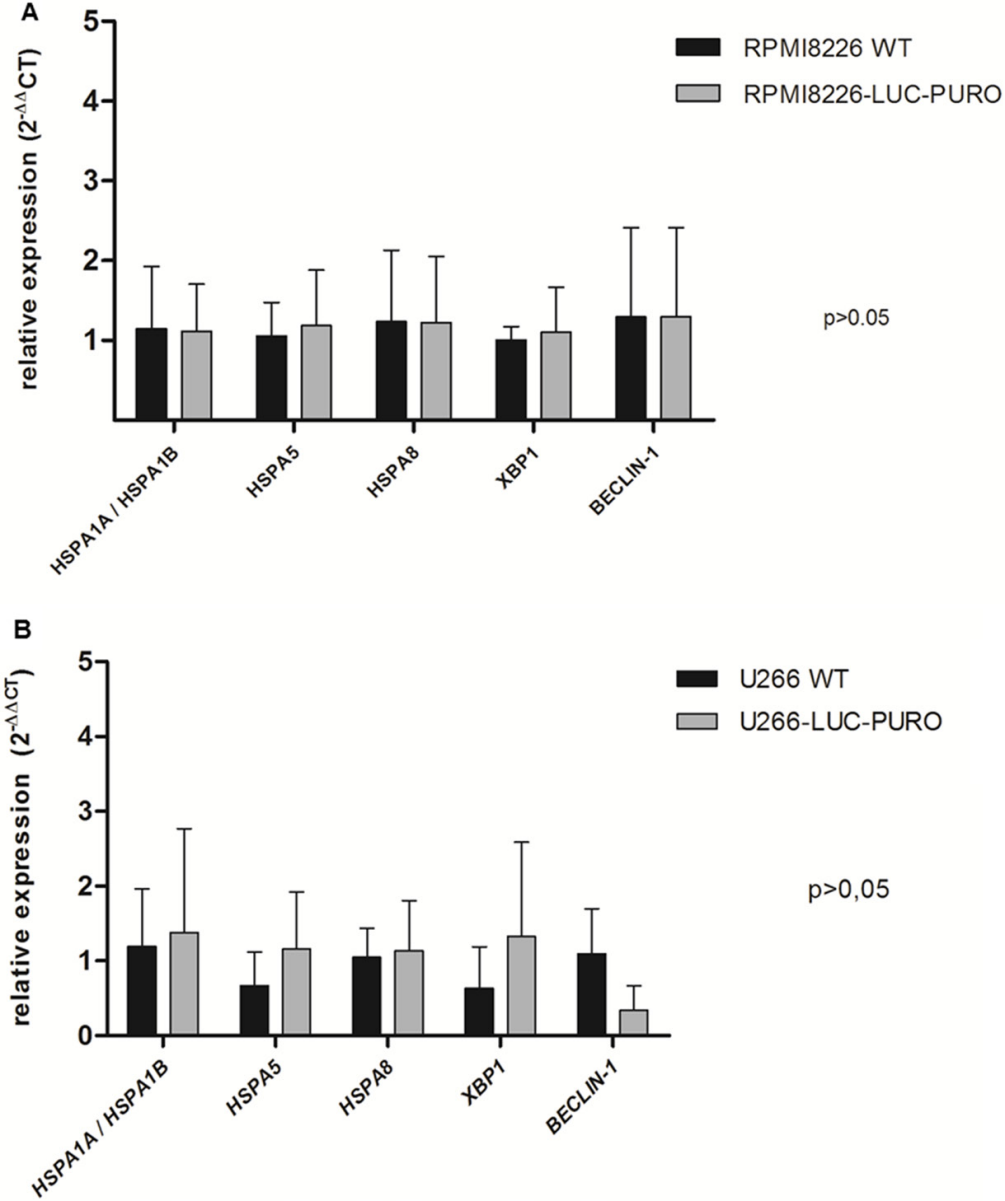
Expression profile of *HSPA1A/HSPA5, HSPA5, HSPA8, XBP-1* and *Beclin-1* genes by real-time quantitative PCR in RPMI8226 (WT) *versus* RPMI8226-LUC-PURO cell lines (**A**) and (**B**) U266 (WT) *versus* U266-LUC-PURO cell lines. Relative expression was performed using 2-^ΔΔCt^ formula and β-actin housekeeping gene. The analyses were done in triplicates. X axis: Genes; Y axis: fold change value. There was no statistically significant difference in the expression of all genes when comparing the two cell lines, with or without transduction with luciferase gene (*p* > 0.05). One-Way ANOVA, with Bonferroni’s post -test.

**Figure 2:**
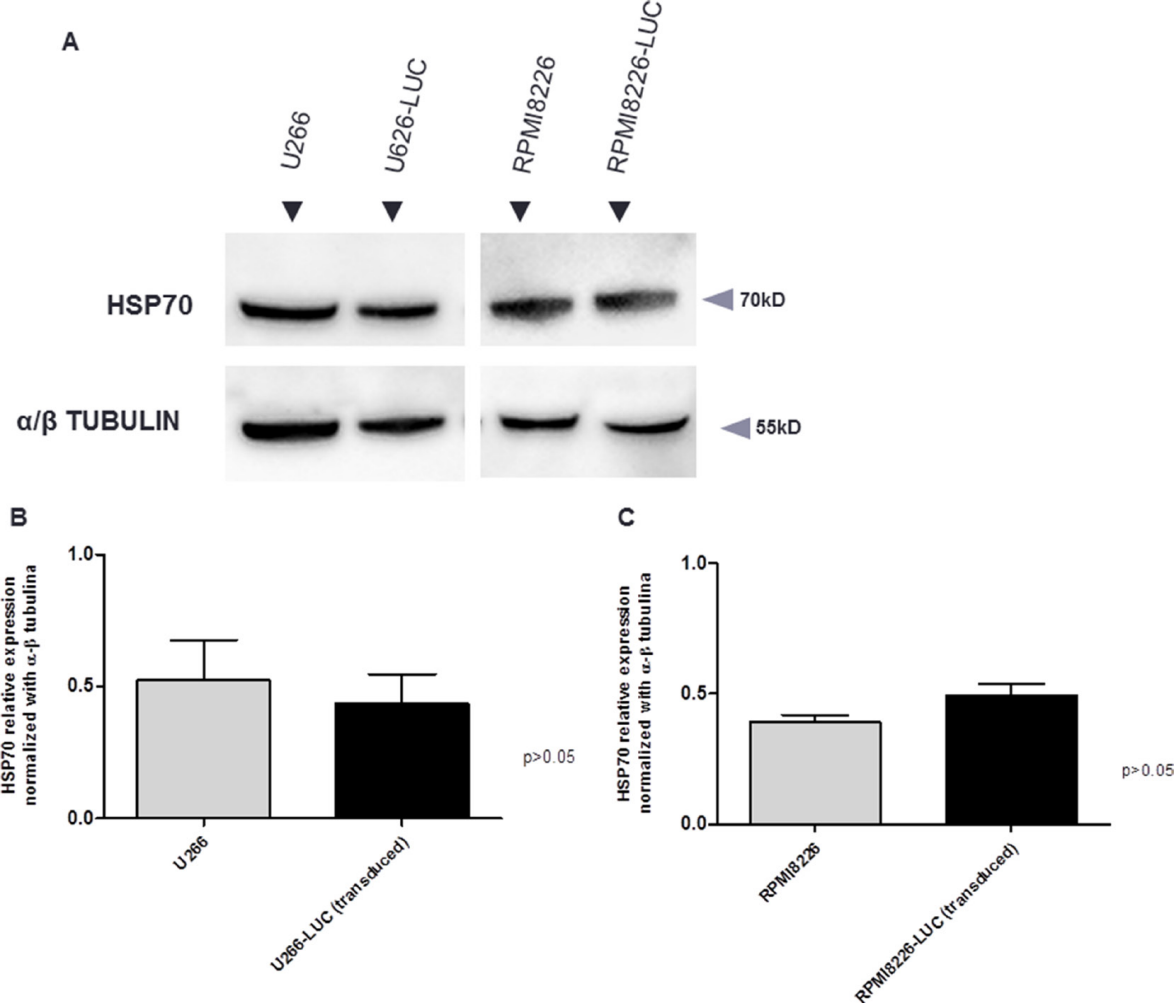
(**A**) Western Blotting analysis. HSP70 protein expression analysis derived from wild type cell lines (U266 and RPMI8226) and transduced cell lines (U266-LUC and RPMI8226-LUC). Densitometry analysis of protein bands. (**B**) U266 *versus* U266-LUC cell line. Graph of HSP70 protein relative expression was normalized with α/β tubulin. (**C**) RPMI8226 *versus* RPMI8226-LUC. Graph of HSP70 protein relative expression was normalized with α/β tubulin. Bands were measured 3 times and represented in the graphs.

RPMI8226-LUC-PURO and U266-LUC-PURO cell lines express HSP70 family genes (*HSPA1A/HSPA1B, HSPA5 and HSPA8*), UPR gene *XBP-1* and autophagy related gene *Beclin-1*. There was no difference in gene expression when bioluminescent and wild type cell lines were compared (Figure 1). HSP70 protein expression was also evaluated comparing bioluminescent and wild type cell lines: there was no statistically significant difference in protein expression for both cell lines after luciferase gene transduction (Figure 2).

**Figure 3:**
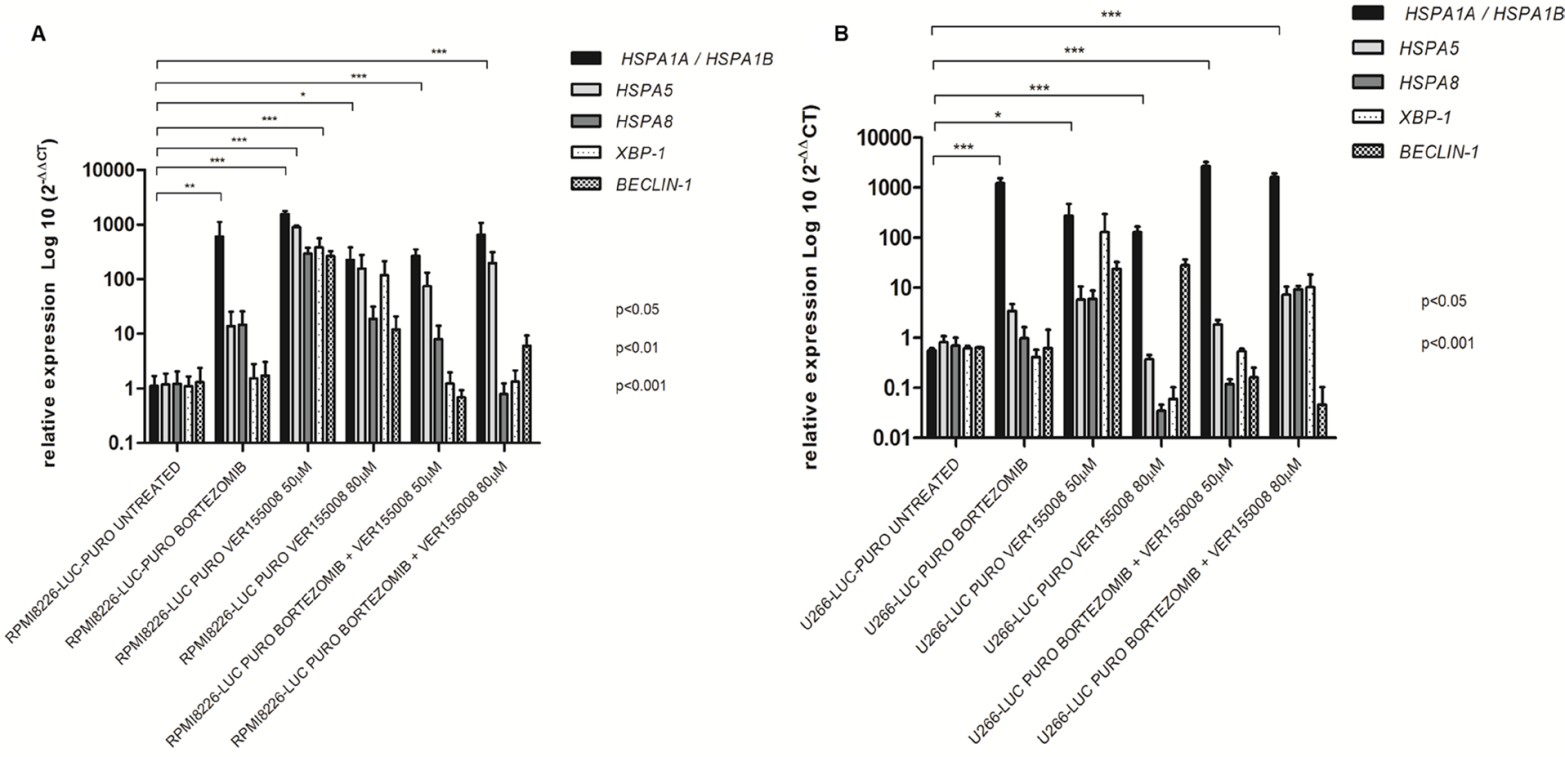
Relative expression of *HSPA1A/HSPA1B, HSPA5, HSPA8, XBP-1* and *Beclin-1* genes in bioluminescent cell lines (**A**) RPMI8226-LUC-PURO untreated *versus* RPMI8226-LUC-PURO treated with bortezomib (100 nM) or VER155008 (50 μM or 80 μM) or bortezomib plus VER155008 (50 μM or 80 μM). (**B**) U266-LUC-PURO untreated *versus* U266-LUC-PURO treated with bortezomib or VER155008 (50 μM or 80 μM) or bortezomib plus VER155008 (50 μM or 80 μM). Relative expression was performed using 2^−DDCt^ formula, with *β*-actin as housekeeping gene and untreated cell line as reference. One-Way ANOVA, with Bonferroni’s post -test.

Treatment with bortezomib and VER155008 (50 μM and 80 μM), isolated or combined, resulted in increased expression of *HSPA1A/HSPA1B* in RPMI8226-LUC-PURO. RPMI8226-LUC-PURO also showed increased expression of *HSPA5* and *XBP-1* after treatment with VER155008 (50 μM) (Figure 3A). U266-LUC-PURO, treated with bortezomib, VER155008 (50μM and 80μM), isolated or combined, responded with increased expression of *HSPA1A/HSPA1B*. There was no statistically significant difference in the expression of other analyzed genes, in comparison to untreated cell line (Figure 3B). Therefore, both cell lines treated with bortezomib, VER155008 (50 μM and 80 μM), isolated or combined, responded with increased expression of *HSPA1A/HSPA1B*.

**Figure 4:**
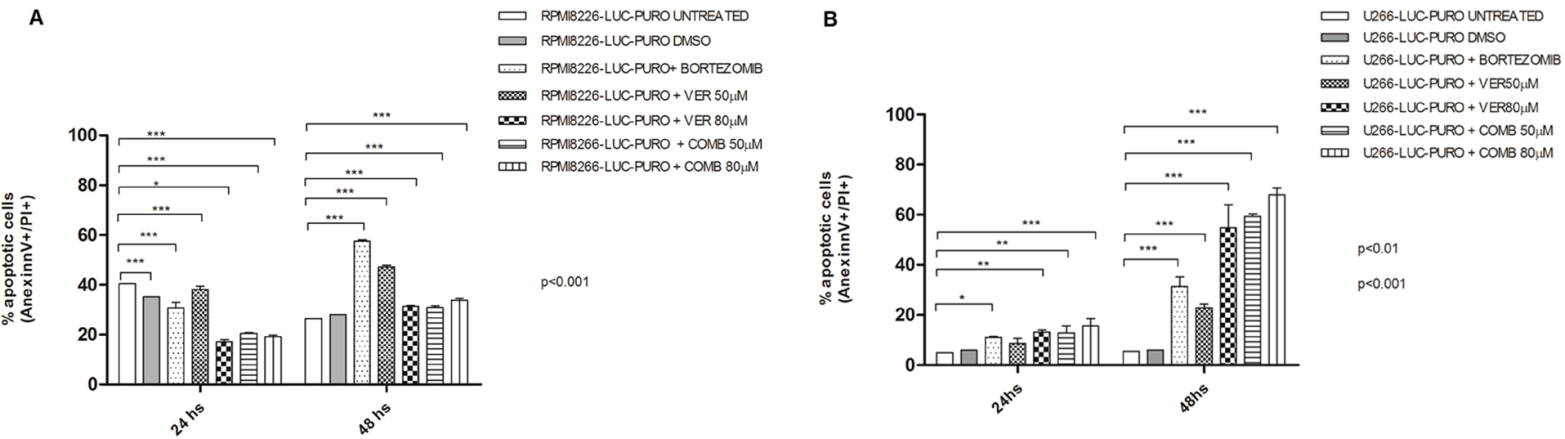
(**A**) Flow cytometry for late apoptosis detection (Annexin V/Propidium iodide) in RPMI8226-LUC-PURO untreated and treated cell line. (**B**) Flow cytometry for late apoptosis detection (Annexin V/Propidium iodide), in U266-LUC-PURO untreated and treated cell line. Analyzes performed 24 and 48 hours after incubation with bortezomib (100 nM) or VER155008 (50 μM or 80 μM) or bortezomib plus VER155008 (50 μM or 80 μM) (named as COMB50 μM or COMB80 μM) for both cell lines. X-axis: time, Y-axis: percentage of cells labeled with annexin V + and PI +. Experiments were performed in triplicates. Two-Way ANOVA, with Bonferroni’s post -test.

*In vitro* studies with RPMI8226-LUC-PURO showed difference in the percentage of cells in apoptosis, over time. There was statistically significant increase in cell death after treatment with bortezomib when compared to controls (*p* < 0.001). Treatment with VER155008 showed a statistically significant difference in cell death when compared to controls (*p* < 0.001). However, none of the above approaches using VER155008, alone or in combination, showed better response comparing with bortezomib alone in this cell line (60% of late apoptosis after 48 hours) (Figure 4A).

U266-LUC-PURO showed good results with all treatments when compared to untreated cell line at 24 and 48 hours (*p* < 0.001) (except for VER155008 concentration 50μM in 24 hours). The percentage of cell death was higher in U266-LUC-PURO treated with VER155008 (50 μM) alone, or treated with VER155008 (80 μM) alone or combined with bortezomib, showing at least 60% of cell death, after 48 hours (Two-way ANOVA, with Bonferroni post-test) (Figure 4B).

**Figure 5:**
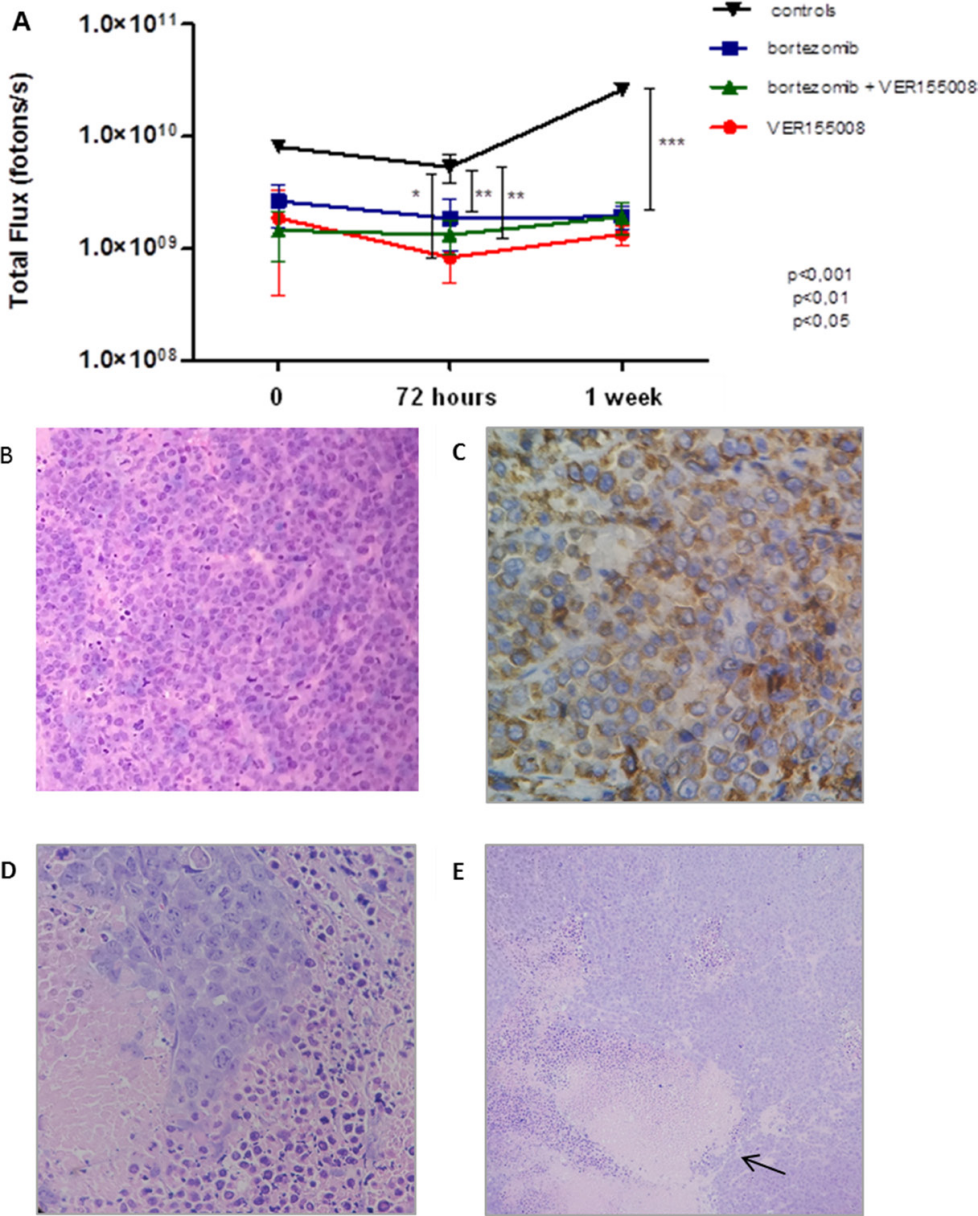
(**A**) Graph: BLI analysis of *Nod.Cg-PrkdcscidII2rgtm-Gammanull* mice. For this analysis, the highest value of bioluminescence acquired for each animal was chosen. (**B**) Histological tumor sample (H and E). Histologic sample obtained from RPMI8226-LUC-PURO tumor tissue of untreated mouse (100 ×). (**C**) Immunohistochemical analysis of the histologic tumor sample. RPMI8226-LUC-PURO tumor tissue of untreated mouse (*lambda* positive) (400 ×). (**D**) Histological tumor sample (H and E). RPMI8226-LUC-PURO tumor tissue from mouse treated with bortezomib plus VER155008 showing tumor coagulative necrosis area and apoptotic cells (400×). (**E**) Histological tumor sample (H&E). The same image above with magnification 100 ×, showing tumor coagulative necrosis area (arrow) and apoptotic cells.

**Figure 6:**
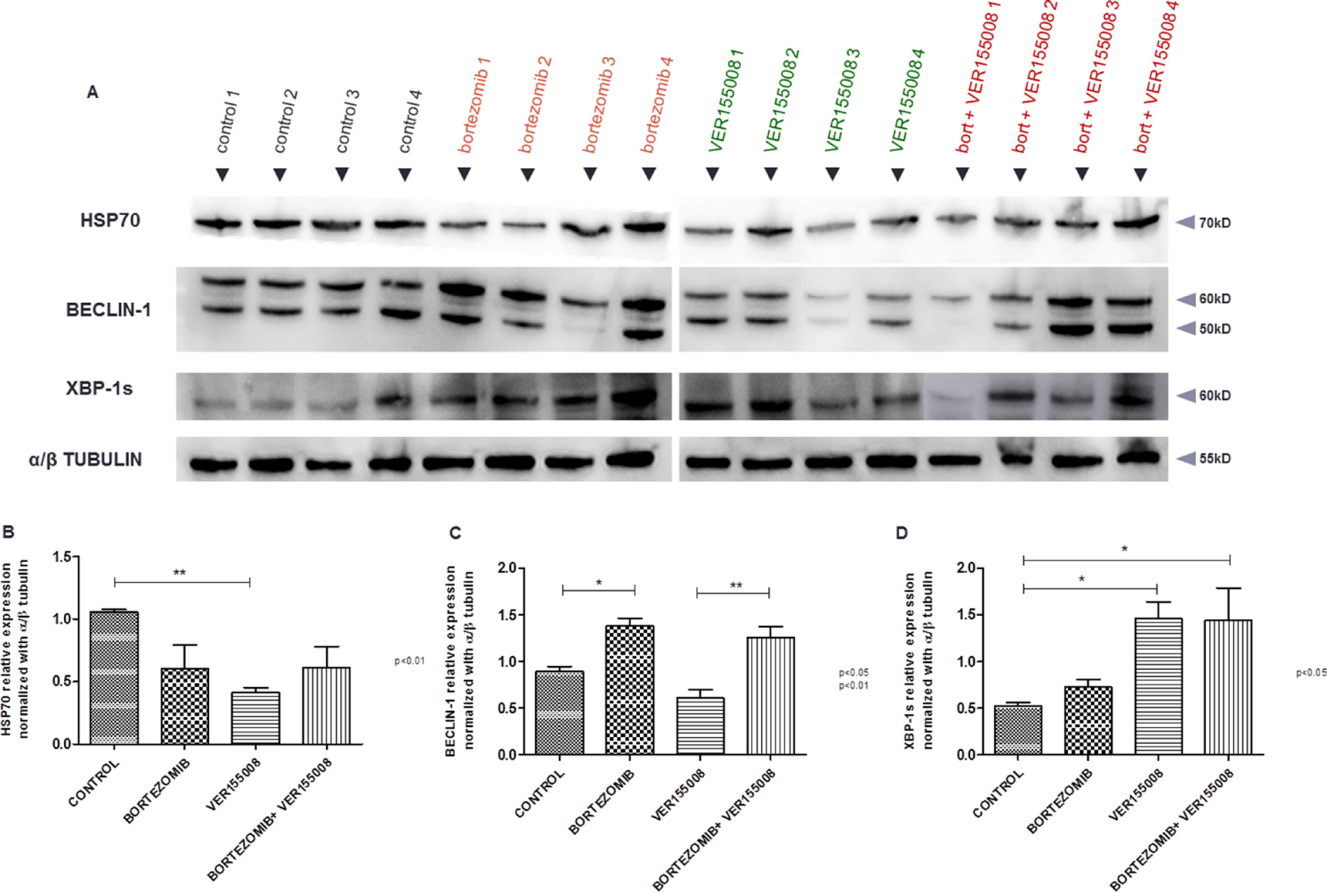
(**A**) Western blotting analysis. HSP70, Beclin-1 and XBP-1s protein expression analysis derived from tumor tissue of untreated *Nod.Cg-PrkdcscidII2rgtm-Gammanull* mice and mice treated with either bortezomib (1 mg/kg) or VER155008 (40 mg/kg), alone or in combination, for one week (*n* = 4 in each group). Densitometry analysis of protein bands. (**B**) Graph of HSP70 protein relative expression normalized with α/β tubulin, calculated using four replicates. (**C**) Graph of Beclin-1 protein relative expression normalized with α/β tubulin, calculated using four replicates. (**D**) Graph of XBP1-s protein relative expression normalized with α/β tubulin, calculated using four replicates.

After BLI analyses, *in vivo* model #1 showed RPMI8226-LUC-PURO tumor reduction in all mice treated with bortezomib, VER155008 or drug combination compared to control group (Figure 5A). Histological analysis from tumors (positive for lambda light chain, Figure 5C) of control mice exhibited a large amount of tumor cells (Figure 5B), whereas the evaluation of the histological sample of mice treated with bortezomib and VER155008 combination demonstrated apoptotic cells areas and coagulative necrosis that may correspond to the results of bioluminescence (cold areas) (Figures 5D and 5E). Expression of HSP70 was reduced in mice treated with all drugs, but it was statistically significant when VER155008 was compared to controls. Beclin-1 showed increased expression in mice treated with bortezomib compared to controls. VER155008 plus bortezomib combination was also able to increase Beclin-1 expression when compared to VER155008 alone. In mice treated with VER155008, isolated or combined with bortezomib, expression of XBP-1s showed increased band intensity when compared to controls (Figure 6). Although the individual results are not homogeneous in all groups for tested proteins, graph bars represent protein relative expression, normalized with α/β tubulin, using four replicates.

**Figure 7:**
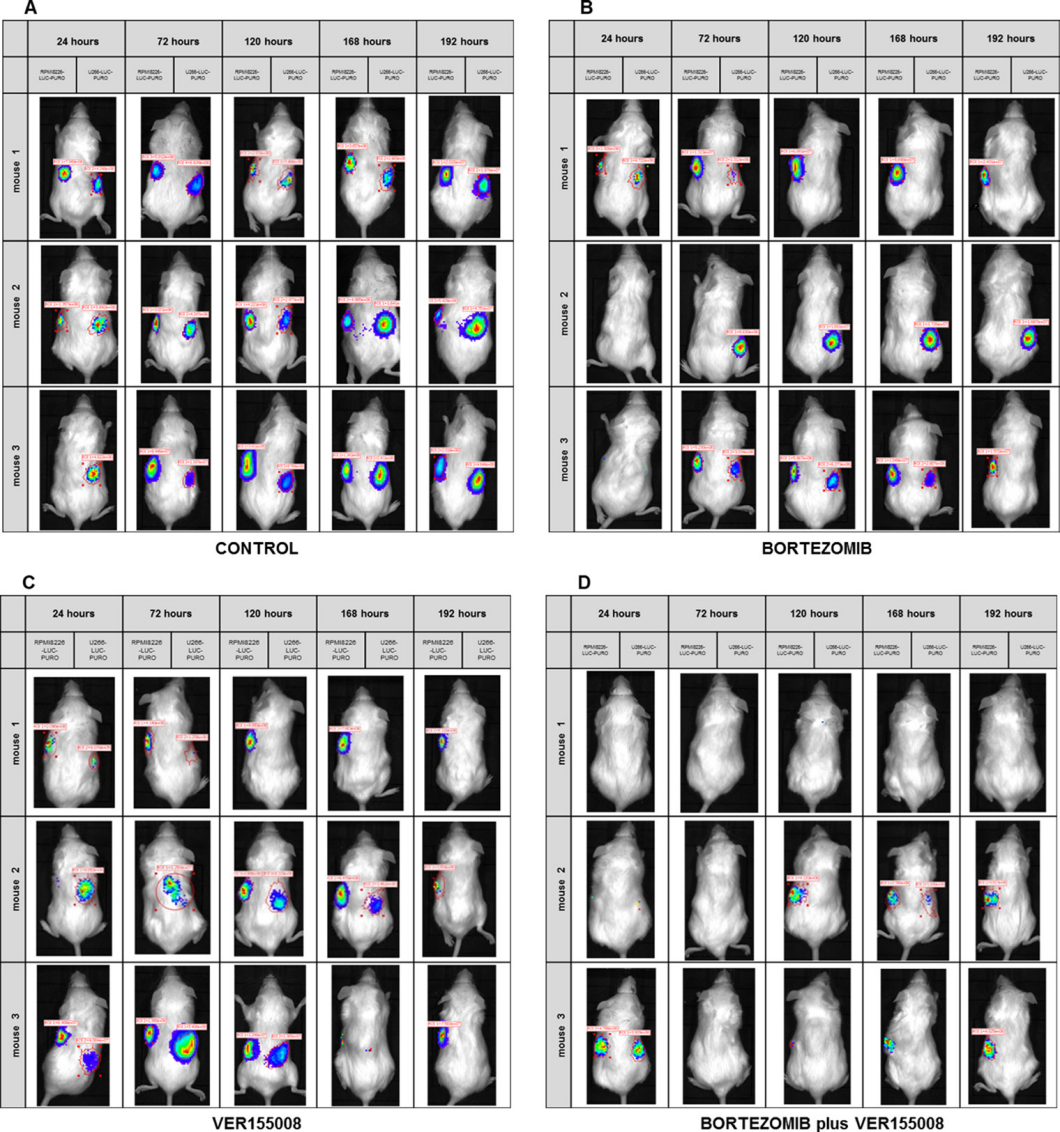
(A–D) BLI analysis of *Nod.Cg-PrkdcscidII2rgtm-Gammanull* mice xenotransplanted with RPMI8226-LUC-PURO cell line (left flank) and U266-LUC-PURO cell line (right flank) treated with either bortezomib (1 mg/kg) or VER155008 (40 mg/kg), alone or in combination, for one week (*n* = 3 in each group). (A) Control group consisted of mice with no intervention. (B) Treatment with bortezomib. (C) Treatment with VER155008. (D) Treatment with bortezomib with VER155008. Evaluations were performed 24, 72, 120, 168 and 192 hours after treatment (triplicate analyzes were performed). BLI acquisition was performed using IVIS Kinetic equipment. (Two-way ANOVA with Bonferroni post- test).

**Figure 8:**
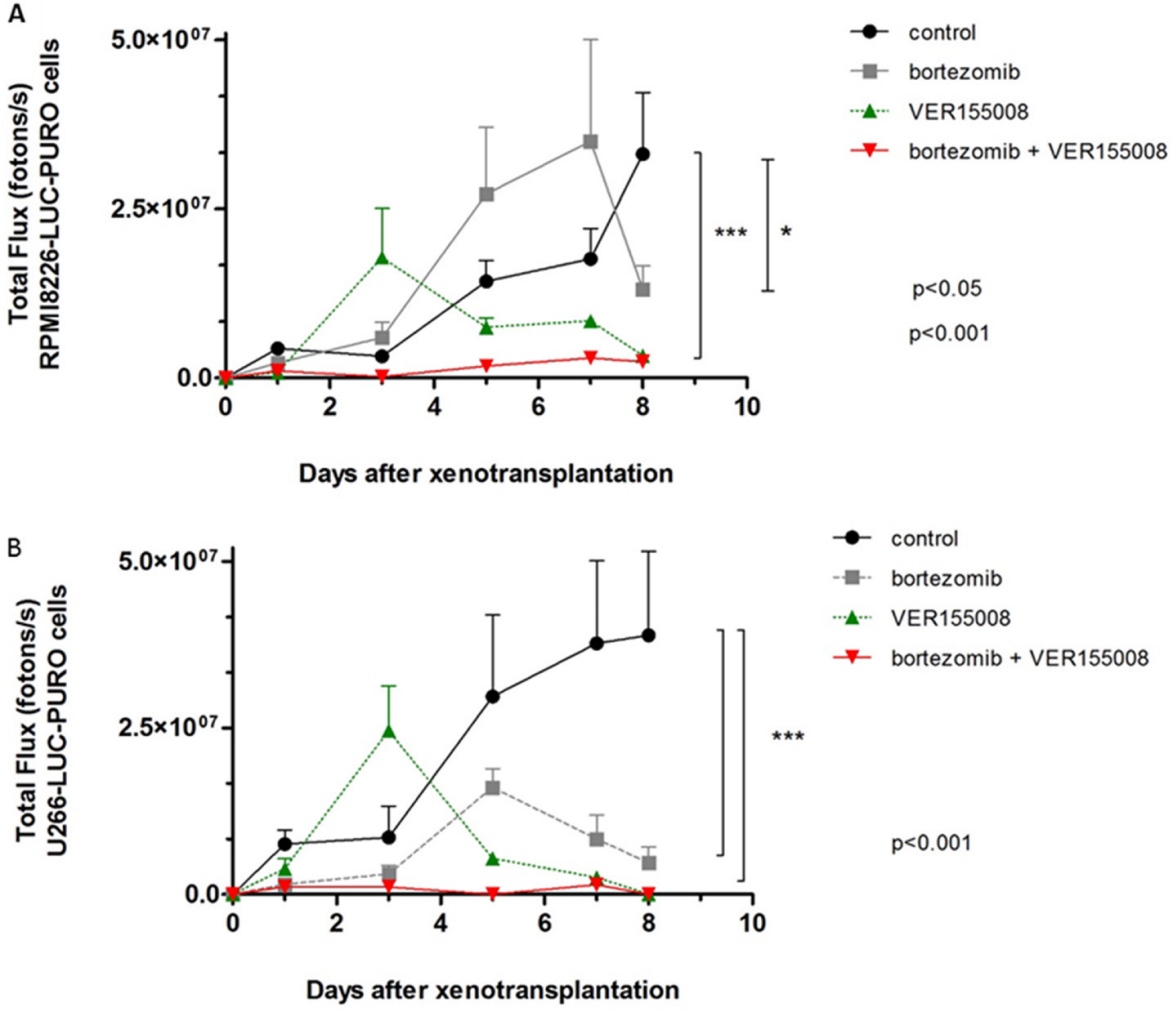
The graphs correspond to the measurement of bioluminescence in *Nod.Cg-PrkdcscidII2rgtm-Gammanull* mice treated with VER155008 (40 mg/kg) or bortezomib (1 mg/kg), alone or in combination, for one week (**A**) BLI analysis of *PrkdcscidII2rgtm-Gammanull* mice related to RPMI8226-LUC-PURO cells xenograft (*n* = 3). Mice treated with VER155008 (40 mg/kg) or treated with bortezomib (1 mg/kg) plus VER155008 showed reduction in bioluminescence comparing to control group (*p* < 0.001), while treatment with bortezomib showed reduction in bioluminescence only after seven days of evaluation (*p* < 0.05). (**B**) BLI analysis of *PrkdcscidII2rgtm-Gammanull* mice related to U266-LUC-PURO cells xenograft (*n* = 3). Mice treated with VER155008 (40 mg/kg) or treated with bortezomib (1 mg/kg) plus VER155008 showed reduction in bioluminescence comparing to control group (*p* < 0.001), while treatment with bortezomib showed reduction in bioluminescence only after seven days of evaluation (*p* < 0.05).

**Figure 9:**
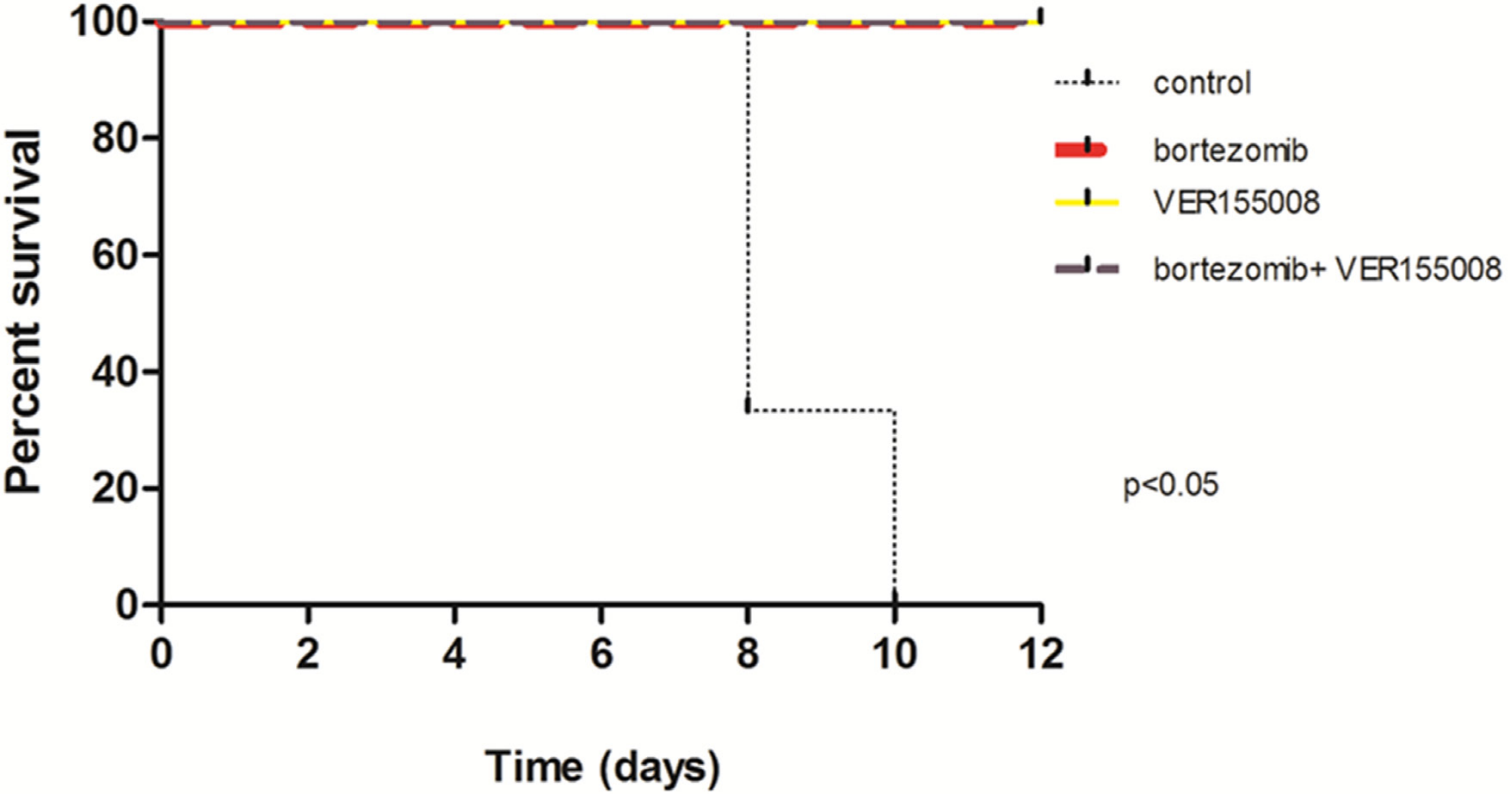
Survival curve of *PrkdcscidII2rgtm-Gammanull* mice xenotransplanted with U266-LUC-PURO and RPMI8226-LUC-PURO *N* = 3 in each group: control, bortezomib, VER155008 and combination of bortezomib and VER155008. The analysis was performed after 12 days of follow up.

In *in vivo* model #2, instead of treating tumors after growth, we treated mice at the same time when cell lines were transplanted. We used RPMI8226-LUC-PURO in the right flank and U266-LUC-PURO in the left flank to compare the behavior of both cell lines in the same mice. There was inhibition (or incipient growth) of tumor growth after treatment with bortezomib, isolated or combined with VER155008 assessed by BLI (Figure 7) after one week, for both cell lines when compared to the control group (Figure 8). Treatment with either bortezomib or VER155008 (isolated or combined) increased mice survival when compared to control group (death of two mice during monitoring), but the difference was not significant (Figure 9).

## DISCUSSION

Since HSP70 connects multiple signaling pathways that work synergistically to protect tumor cells from death by proteotoxic stress, it can represent a key role to establish a new approach for MM treatment. In this study, we evaluated the combination of a proteasome inhibitor (bortezomib) with a HSP70 inhibitor (VER155008) to understand how pathways related to protein homeostasis behave (heat shock proteins, UPR, ubiquitin-proteasome system and autophagy) when we interfere in two important processes for myeloma cells survival. Our study shows that combination of proteasome and HSP70 inhibitors induced cell death in tumor cells *in vitro* (late apoptosis induction) and *in vivo* (inhibition of tumor growth).

The rationale for our study was also used by Braunstein *et al.* (2011) [19]. The study demonstrated that the combination of MAL3-101, a HSP70 inhibitor, with a proteasome inhibitor (MG-132) may reduce the resistance of myeloma cells to proteasome inhibitors. In our study, we used two multiple myeloma cell lines recognized for the resistance or sensitivity to bortezomib (U266 and RPMI8226, respectively), in an attempt to confirm this action [19, 28].

In addition to exploring the role of HSP70 family in MM, our study showed how mechanisms related to protein homeostasis behave after inhibition of HSP70 and ubiquitin proteasome pathway, through evaluation of *XBP-1* and *Beclin-1*.

RPMI8226-LUC-PURO and U266-LUC-PURO cell lines express HSP70 family genes (*HSPA1A/HSPA1B, HSPA5 and HSPA8*), UPR gene *XBP-1* and autophagy related gene *Beclin-1* and there was no difference in gene expression when bioluminescent and wild type cell lines were compared, making the bioluminescent cell suitable for this study. Additionally, HSP70 protein expression was similar in bioluminescent and wild type cells by Western Blotting analysis. Protein bands were quantified by densitometry and showed no statistically significant difference between bioluminescent and wild type cells (Figure 2), confirming qPCR results.

*HSPA1A/HSPA1B* showed increased expression when both bioluminescent cell lines were treated with bortezomib for 24 hours (isolated or associated with VER155008), suggesting that the inhibition of the proteasome - which acts to degrade and eliminate ubiquitinated proteins - by bortezomib requires intense activity of HSP70 as a compensation mechanism to avoid proteotoxic stress and cell death [29]. The isolated use of VER155008 at concentrations of 50μM and 80μM, for both cell lines, also led to a statistically significant increase in *HSPA1A/HSPA1B* expression, suggesting that competitive inhibition of the HSP70 binding site by VER155008 should also result in an increased expression of these genes as a compensation mechanism after treatment, which induce cellular stress. *HSPA1A/HSPA1B* and *HSPA8* members share 85% sequence homology, and are considered to have compensatory roles [11, 15].

Bortezomib used as single agent was able to induce late apoptosis in RPMI8226-LUC-PURO. On the other hand, U266-LUC-PURO presented a higher percentage of apoptosis when treated with bortezomib plus VER155008 drug combination, overcoming the response to bortezomib alone.

U266 cell line is recognized for being resistant to bortezomib [28], but combination of HSP70 inhibitor with bortezomib was able to overcome drug resistance and induced apoptosis, showing the synergistic effects of drug combination for U266-LUC-PURO: our study showed that bortezomib induced only 35% of cell death after 48 hours of incubation; treatment with VER155008 (80 μM) induced more than 60% of cell death, and bortezomib plus VER155008 combination induced more than 70% of cell death. Zhang *et al.* (2013) [11] also demonstrated that treatment with VER155008 (80 μM) isolated for 24 hours resulted in higher percentage (60%) of cell death in U266 cell line.

A possible explanation to different results in both cell lines relies on genetic background. RPMI8226 presents t(14;16) translocation, *TP53* point mutation [located in exon 7 (c.853G>A), resulting in a *TP53* missense mutation (p.E285K)], and no *TP53* deletion by FISH [30]. U266 cell line has t(11;14), E419X missense mutation on *RB1* and A161T missense mutation on *TP53* and also deletion of 17p [30, 31]. Therefore, U266-LUC-PURO cell line presented better results with drug combination than with bortezomib alone, despite the high risk genetic profile, including no*TP53* gene activity.

VER155008 is an ATP competitive inhibitor and acts on *HSPA1A/HSPA1B*, *HSPA5* and *HSPA8* in a dose dependent manner [32]. Zhang *et al.* (2013) [11] tested VER155008 in four myeloma cell lines (U266, RPMI8226, H929 and KMS11), using concentrations between 5 to 80 μM, for 24 hours, and showed that higher concentrations of VER155008 led to increased cell death rates. These results were confirmed by our group using RPMI8226 and U266 cell lines, since we observed higher cell death rates with 50 μM and 80 μM of VER155008, respectively.

For our *in vivo* model #1, treatment with bortezomib, VER155008 or drug combination demonstrated reduced tumor growth after seven days with any of the drugs. This reduction in tumor growth was identified by decrease in bioluminescence, but not in tumor size, compared to control group (data not shown). Presumably, high sensitivity of BLI identifies active tumor areas versus necrotic areas (blue and purple signals in BLI) and tumor measurement does not take into consideration necrotic areas (Figure 4D and 4E). Mice RPMI8226-LUC-PURO tumors treated with bortezomib or VER155008, isolated or combined, presented decreased HSP70 protein expression; as expected, treatment with VER155008 alone led to a statistically significant protein expression reduction comparing to controls (*p* < 0.01). Increased expression of Beclin-1 in mice tumors treated with bortezomib, isolated or combined with VER155008, suggests that inhibition of the proteasome and/or HSP70 increases autophagy as a compensatory mechanism. Expression of XBP1-s (which is the functionally active transcription factor for UPR) in mice tumors treated with VER155008, isolated or combined with bortezomib, showed increased band intensity when compared to controls, also suggesting that UPR activity was used to avoid ER stress due to impairment of related pathways involved in protein homeostasis.

In our *in vivo* model #2, we treated mice at the same time when cell lines were transplanted. With this strategy, treatment with drugs alone (especially VER155008) or in combination (VER155008 and bortezomib) demonstrated significant impact in preventing tumor growth for both RPMI8226-LUC-PURO and U266-LUC-PURO, compared to the untreated group.

In conclusion, our study showed that the combination of proteasome and HSP70 inhibitors induced cell death in tumor cells, with particular relevant results in U266 cell line, which presents high risk genetic profile. Since HSP70 connects multiple signaling pathways, it works synergistically to protect tumor cells from death by proteotoxic stress, and can represent a key role to establish a new approach for high risk MM treatment, especially after achievement of the best therapeutic response, or as maintenance therapy.

## MATERIALS AND METHODS

### Ethical aspects

The present study was approved by the Federal University of São Paulo Ethical Committee (CEP/CEUA 0219/2012).

### Cell lines

MM cell lines RPMI8226 and U266 were maintained in culture medium (RPMI-1640 supplemented with 10% fetal bovine serum, 1% L-glutamine, 1% NEAA [non essentials amino acids] – Gibco Laboratory, Grand Island, NY, USA – and garamicine), at 37°C with 5% CO_2_.

### Lentiviral transduction

1 × 10^6^ RPMI8226 and U266 cells were transduced with a luciferase expression vector (*p-Lenti PGK V5-LUC Puro*, *Firefly luciferase,* Addgene, Cambridge, MA, USA) at a multiplicity of infection of 0.5 in 600 μL of RPMI 1640 culture medium with L-glutamine (Life Technologies, Carlsbad, CA, USA), supplemented with fetal bovine serum (10%), NEAA and 8μg/mL of Polibrene (Sigma Aldrich Co., St Louis, MO, USA). Medium with viral particles was replaced after 8 hours and cells were maintained in culture for 3 weeks in the same culture medium plus 2 μg/mL of puromycin (selecting permanent expression of transduced genes). After this step, the bioluminescent cells were named RPMI8226-LUC-PURO and U266-LUC-PURO.

### RNA extraction

RNA was extracted from MM cell lines using TRIzol reagent (Invitrogen, Carlsbad, CA, USA), following the manufacturer’s instructions. RNA was purified with phenol and chloroform and its integrity was evaluated by 1% agarose gel electrophoresis.

### RNA quantification and cDNA synthesis

RNA was quantified by DS-11 spectrophotometer (Denovix, Thermo, Rockford, IL, USA). 25μg of RNA were treated with RQ1 DNase (Promega Corporation, Madison, WI, USA), following manufacturer’s instructions. 2 μg of total RNA were reverse transcribed with *SuperScript III* (Invitrogen, Carlsbad, CA, USA), according to manufacturer’s instructions.

### Real time quantitative PCR (qPCR)

Gene expression analyses were performed in RPMI8226-LUC-PURO and U266-LUC-PURO cell lines, with or without treatment with bortezomib (100 nM) or VER155008 (in two concentrations 50 μM or 80 μM), isolated or combined, using the 7500 Real Time PCR System (Applied Biosystems, Foster City, CA, USA). β-actin was used as housekeeping gene for *HSP70* family members (Hs00271244_s1 for *HSPA1B* and *HSPA1A*; HS00607129_gh for *HSPA5* and Hs03044880_gh for *HSPA8*), autophagy and UPR related genes (Hs00186838_m1 for *Beclin-1(BECN1)* and Hs00231936_m1 for *XBP-1*, respectively) (for all genes, Life Technologies, Carlsbad, CA, USA). Relative gene expression was calculated using the equation 2^−DDCt^ [21].

### Apoptosis analyses

For apoptosis assays, bortezomib (100nM) and VER155008 (50 μM or 80 μM) [11] (both from Selleckchem, Huston, TX, USA) were used in the bioluminescent cell lines (1 × 10^5^ RPMI8226-LUC-PURO and U266-LUC-PURO), isolated or combined. Cells were analyzed in triplicate after 24 and 48 hours of treatment. Two controls were used: cells without any drug treatment and treated with DMSO (drug diluent, for both drugs bortezomib and VER155008). Cells were labeled with annexin V and/or propidium iodide (PI) (Becton Dickinson, Franklin Lakes, NJ, USA), according to manufacturer’s instructions.Fluorescence intensity was evaluated with the cytometer *FACSCalibur* (BD Biosciences, Franklin Lakes, NJ, USA).

### Xenograft model #1: tumor growth reduction

*NOD.Cg-PrkdcscidII2rgtm-Gammanull* female mice (*n* = 16) (6-to-8-week age) were inoculated subcutaneously into the right flank with 1 × 10^6^ RPMI8226-LUC-PURO cells together with 200 μL of matrigel basement membrane matrix (BD Bioscience, Bedford, MA). When tumor achieved between 50 mm^3^ to 80 mm^3^ in diameter, mice were randomized into four treatment groups (*n* = 4 in each group), receiving intravenous bortezomib (1 mg/kg), or VER155008 (40 mg/kg), or bortezomib plus VER155008 combination [22]. The control group consisted of mice without intervention. Mice were treated for one week, being VER155008 administered as a single dose and bortezomib given twice a week. Evaluations were performed before treatment (point 0), after 72 hours and after one week of treatment. Bioluminescence acquisition (total flux, fotons/s) was performed by the IVIS Kinetic equipment (triplicate analyses were performed for each animal) [23, 24]. Each photon is represented by a number equivalent to its intensity. A color is associated with each numeric value and a color scale is created. The amount of light emitted was evaluated in photons/second (photons/s). The photon measurement represents a calibrated unit of photon emission through the sample and is used to quantify bioluminescence according to the Living Image Software User´s Manual (Capiler Life Science, Hopkinton, MA, USA). Mice received intraperitoneal luciferin injection (150 mg/kg) 10 minutes before the procedure and after isoflurane anesthesia (1 mg/kg) [25–27]. Tumors were resected and frozen at −80°C for further protein extraction. Tumor specimens were also analyzed in microscope after hematoxylin/eosin and light chain stains.

### Xenograft model #2: tumor growth inhibition

*NOD.Cg-PrkdcscidII2rgtm-Gammanull* female mice (*n* = 12) (6-to-8-week age) (divided into four treatment groups, containing 3 mice each), were treated immediately after transplant of the cell lines together with 200μL of matrigel basement membrane matrix (U266-LUC-PURO on the right flank and RPMI8226-LUC-PURO on the left flank). Treatment was performed in the same way described for model #1. Control group consisted of mice without intervention. Evaluations were performed 24, 72, 120, 168 and 192 hours after treatment. The acquisition of bioluminescence was performed in the same way described for model #1.

### Western blotting

Proteins were extracted from cell lines or frozen tumors (−80°C) using the Total Protein extraction kit (RF-2140) (EDM-Millipore, Darmstadt, Germany) according to manufacturer’s recommendations. Protein quantification was performed with DS-11 spectrophotometer (Denovix, Thermo, Rockford, IL, USA), using the Bradford method. 20 μg (cell lines) or 10 μg (tumors) of protein were used in 1% polyacrylamide gel electrophoresis containing sodium dodecyl sulfate (SDS-PAGE) for band separation. After electrophoresis, proteins were transferred to a polyvinylidene fluoride (PVDF) membrane (Amersham Hybond-P, GE Healthcare, Buckinghamshire, UK). Next, membranes were incubated overnight at 4°C with anti-HSP70 (HSP70 antibody detects endogenous levels of total HSP70 protein and HSC70), anti-XBP-1s, anti-Beclin-1 or anti-α/β (control) primary antibody (Cell Signaling, Danvers, MA, USA) (dilution - 1:1000). After incubation with secondary antibody, *Immobilion Western Chemiluminescent HRP Substrate* (Millipore, Darmstadt, Germany) was used in the proportion of 1:1 to highlight protein presence. The membrane was visualized on an UVITEC Cambridge equipment (UVItec Limited, Cambridge, UK) with chemiluminescence filter. D*ensitometric* analysis was performed to quantify bands volume using UVITEC BAND software (UVItec Limited, Cambridge, UK). The ratios were calculated using the respective α/β-tubulin bands as normalizers.

### Statistical analysis

Gene expression in luciferase transduced cell lines was analyzed with One-Way ANOVA, followed by Bonferroni’s post-test. The other comparisons between the different treatment groups (*in vitro* and *in vivo* studies), over time, were performed with Two-way ANOVA test, and Bonferroni post-test, using Graphpad Prism 5 software. Values of *p* < 0.05 were considered significant.

## References

[1] Palumbo A, Anderson K (2011). Multiple Myeloma. New Engl J of Med.

[2] Nooka A, Kastritis E, Dimopoulos M, Lonial S (2015). Treatment options for relapsed and refractory multiple myeloma. Blood.

[3] Mimura N, Hideshima T, Anderson K (2015). Novel therapeutic strategies for multiple myeloma. Exp Hemat.

[4] Mitsiades C (2015). Therapeutic landscape of carfilzomib and other modulators of the ubiquitin-proteasome pathway. J Clin Oncol.

[5] Moreau P, Attal M, Facon T (2015). Frontline therapy of multiple myeloma. Blood.

[6] Rajkumar VS (2017). Treatment Guidelines: Multiple Myeloma. Treatment of Newly Diagnosed Myeloma.

[7] Felix R, Colleoni GW, Caballero OL, Yamamoto M, Almeida MS, Andrade VC, Chauffaille Mde L, Silva WA, Begnami MD, Soares FA, Simpson AJ, Zago MA, Vettore AL (2009). SAGE analysis highlights the importance of P53CSV, DDX5, MAPKAPK2 and RANBP2 to multiple myeloma tumorigenesis. Cancer Letters.

[8] Park W (2005). P53CSV, a novel p53-inducible gene involved in the p53-dependent cell-survival pathway. Cancer Research.

[9] Fook-Alves VL, de Oliveira MB, Zanatta DB, Strauss BE, Colleoni GW (2016). TP53 Regulated Inhibitor of Apoptosis 1 (TRIAP1) stable silencing increases late apoptosis by upregulation of caspase 9 and APAF1 in RPMI8226 multiple myeloma cell line. Biochim Biophys Acta.

[10] Murphy M (2013). The HSP70 family and cancer. Carcinogenesis.

[11] Zhang L, Fok J, Mirabella F, Aronson L, Fryer R, Workman P, Morgan GJ, Davies FE (2013). HSP70 inhibition induces myeloma cell death via the intracellular accumulation of immunoglobulin and the generation of proteotoxic stress. Cancer Letters.

[12] Aronson L, Davies F (2012). DangER: protein ovERload. Targeting protein degradation to treat myeloma. Haematologica.

[13] Nikesitch N, Ling S (2015). Molecular mechanisms in multiple myeloma drug resistance. J of Clin Pathol.

[14] Vincenz L, Jager R, O'Dwyer M, Samali A (2013). Endoplasmic reticulum stress and the unfolded protein response: targeting the Achilles heel of multiple myeloma. Mol Cancer Ther.

[15] Zhang L, Fok J, Davies FE (2014). Heat shock proteins in multiple myeloma. Oncotarget.

[16] Jolly C (2000). Role of the heat shock response and molecular chaperones in oncogenesis and cell death. J Natl Cancer Inst.

[17] Syrigos K, Harrington K, Karayiannakis A, Sekara E, Chatziyianni E, Syrigou E, Waxman J (2003). Clinical significance of heat shock protein-70 expression in bladder cancer. Urology.

[18] Calderwood S, Khaleque M, Sawyer D, Ciocca D (2006). Heat shock proteins in cancer: chaperones of tumorigenesis. Trends Biochem Sci.

[19] Braunstein M, Scott S, Scott C, Behrman S, Walter P, Wipf P, Coplan JD, Chrico W, Joseph D, Brodsky JL, Batuman O (2011). Antimyeloma effects of the heat shock protein 70 molecular chaperone inhibitor MAL3-101. J Oncol.

[20] Ishii T, Seike T, Nakashima T, Juliger S, Maharaj S, Soga S, Akinaga S, Cavenagh J, Joel S, Shiotsu Y (2012). Anti-tumor activity against multiple myeloma by combination of KW-2478, an HSP90 inhibitor, with bortezomib. Blood Cancer J.

[21] Livak K, Schmittgen T (2001). Analysis of relative gene expression data using real-time quantitative PCR and the 2−ΔΔCT method. Methods.

[22] Mitsiades C, Anderson K, Carrasco D (2007). Mouse models of human myeloma. Hematol Oncol Clin North Am.

[23] Swift B, Williams B, Kosaka Y, Wang X, Medin J, Viswanathan S, Martinez-Lopez J, Keating A (2012). Natural killer cell lines preferentially kill clonogenic multiple myeloma cells and decrease myeloma engraftment in a bioluminescent xenograft mouse model. Haematologica.

[24] Tiffen J, Bailey C, Ng C, Rasko J, Holst J (2010). Luciferase expression and bioluminescence does not affect tumor cell growth in vitro or in vivo. Mol Cancer.

[25] Fryer R, Graham T, Smith E, Walker-Samuel S, Morgan G, Robinson SP, Davies FE (2013). Characterization of a novel mouse model of multiple myeloma and its use in preclinical therapeutic assessment. PLoS One.

[26] Bannerman B, Xu L, Jones M, Tsu C, Yu J, Hales P, Monbaliu J, Fleming P, Dick L, Manfredi M, Claiborn C, Bolen J, Berger A (2011). Preclinical evaluation of the antitumor activity of bortezomib in combination with vitamin C or with epigallocatechin gallate, a component of green tea. Cancer Chemother Pharmacol.

[27] Mezzanotte L, Fazzina R, Michelini E, Tonelli R, Pession A, Branchini B, Roda A (2009). In Vivo Bioluminescence Imaging of Murine Xenograft Cancer Models with a Red-shifted Thermostable Luciferase. Mol Imaging Biol.

[28] Park J, Bae E, Lee C, Choi J, Jung WJ, Ahn K, Yoon SS (2014). Establishment and characterization of bortezomib-resistant U266 cell line: Constitutive activation of NF-κB-mediated cell signals and/or alterations of ubiquitylation-related genes reduce bortezomib-induced apoptosis. BMB Reports.

[29] Shah S, Lonial S, Boise L (2015). When cancer fights back: multiple myeloma, proteasome inhibition, and the heat-shock response. Mol Cancer Res.

[30] Myeloma Cell Lines (2016). Common Genetics - Keats Lab. http://www.keatslab.org.

[31] Fernando R, Carvalho F, Mazzotti D, Evangelista A, Braga W, Chauffaille Mde L, Leme AFP, Colleoni GWB (2015). Multiple myeloma cell lines and primary tumors proteoma: protein biosynthesis and Immune system as potential therapeutic targets. Genes Cancer.

[32] Schlecht R, Scholz S, Dahmen H, Wegener A, Sirrenberg C, Musil D, Bomke J, Eggenweiler H, Mayer MP, Bukau B (2013). Functional Analysis of Hsp70 Inhibitors. PLoS One.

